# Advanced glycation end products induce cell cycle arrest and proinflammatory changes in osteoarthritic fibroblast-like synovial cells

**DOI:** 10.1186/ar2807

**Published:** 2009-09-07

**Authors:** Sybille Franke, Manfred Sommer, Christiane Rüster, Tzvetanka Bondeva, Julia Marticke, Gunther Hofmann, Gert Hein, Gunter Wolf

**Affiliations:** 1 Department Internal Medicine III, Jena University Hospital, Erlanger Allee 101, Jena, 07740, Germany; 2 Department of Traumatology, Hand and Reconstructive Surgery, Jena University Hospital, Erlanger Allee 101, Jena, 07740, Germany

## Abstract

**Introduction:**

Advanced glycation end products (AGEs) have been introduced to be involved in the pathogenesis of osteoarthritis (OA). The influence of AGEs on osteoarthritic fibroblast-like synovial cells (FLS) has been incompletely understood as yet. The present study investigates a potential influence of AGE-modified bovine serum albumin (AGE-BSA) on cell growth, and on the expression of proinflammatory and osteoclastogenic markers in cultured FLS.

**Methods:**

FLS were established from OA joints and stimulated with AGE-BSA. The mRNA expression of p27^Kip1^, RAGE (receptor for AGEs), nuclear factor kappa B subunit p65 (NFκB p65), tumor necrosis factor alpha (TNF-α, interleukin-6 (IL-6), receptor activator of NFκB ligand (RANKL) and osteoprotegerin was measured by real-time PCR. The respective protein expression was evaluated by western blot analysis or ELISA. NFκB activation was investigated by luciferase assay and electrophoretic mobility shift assay (EMSA). Cell cycle analysis, cell proliferation and markers of necrosis and early apoptosis were assessed. The specificity of the response was tested in the presence of an anti-RAGE antibody.

**Results:**

AGE-BSA was actively taken up into the cells as determined by immunohistochemistry and western blots. AGE-induced p27^Kip1 ^mRNA and protein expression was associated with cell cycle arrest and an increase in necrotic, but not apoptotic cells. NFκB activation was confirmed by EMSAs including supershift experiments. Anti-RAGE antibodies attenuated all AGE-BSA induced responses. The increased expression of RAGE, IL-6 and TNF-α together with NFκB activation indicates AGE-mediated inflammation. The decreased expression of RANKL and osteoprotegerin may reflect a diminished osteoclastogenic potential.

**Conclusions:**

The present study demonstrates that AGEs modulate growth and expression of genes involved in the pathophysiological process of OA. This may lead to functional and structural impairment of the joints.

## Introduction

Osteoarthritis (OA) is the most common joint disease of middle aged and older people across the world. OA is caused by joint degeneration, a process that includes progressive loss of articular cartilage accompanied by remodelling and sclerosis of subchondral bone, and osteophyte formation. Currently, the pathophysiology of joint degeneration that leads to the clinical syndrome of OA remains poorly understood [1]. Multiple factors for OA initiation and progression have been identified. These factors can be segregated into categories that include hereditary factors, mechanical factors and effects of ageing [2]. Among these, the most important risk factor is age.

In contrast to rheumatoid arthritis (RA), OA is defined as a non-inflammatory arthropathy, due to the absence of neutrophils in the synovial fluid and the lack of systemic manifestations of inflammation. However, morphological changes found in patients with OA include cartilage erosion as well as a variable degree of synovial inflammation. Proinflammatory cytokines have been implicated as important mediators in the disease [2-4]. Fibroblast-like synovial cells (FLS) are involved in osteoarthritic synovial inflammation. FLS activated by proinflammatory cytokines such as TNF-α and IL-1 show marked increases in the release of matrix metalloproteinases that can promote cartilage degradation [5]. On the other hand, FLS itself may be a source of proinflammatory cytokines [6,7].

Increasing age is accompanied by tissue accumulation of advanced glycation end products (AGEs). AGEs are chemical modifications of proteins by carbohydrates, including metabolic intermediates generated during the Maillard reaction, which are formed during ageing as a physiological process [8].

Metabolic intermediates accumulate in human articular cartilage and bone through life, and affect biomechanical, biochemical and cellular characteristics of the tissues [9,10]. AGEs bind to specific proteins. Among these the 'receptor for AGEs', RAGE, a multiligand member of the immunoglobulin superfamily, is the most well known. Today RAGE is considered to be a pattern recognition receptor. RAGE-ligand interaction results in a rapid and sustained cellular activation of nuclear factor kappa B (NFκB), accompanied by subsequent transcription of proinflammatory cytokines and increased expression of the receptor itself [11,12].

As suggested recently, OA synovitis can be considered to be a common final pathway in a tissue that is easily primed for innate immune responses triggered by cartilage damage [13,14]. In this context, release of AGE-modified molecules from damaged tissue into the synovium may play a role in the initiation and perpetuation of inflammation and degradation processes. RAGE as well as AGEs are present in the synovial lining, sublining and endothelium of OA synovial tissue [15,16]. FLS obtained from patients with OA express RAGE and stimulation of these cells with AGEs upregulates metalloproteinases [17].

For FLS obtained from patients with RA, it was shown that intraarticular serum amyloid A, which is also a RAGE ligand, could activate NFκB signalling through binding to cell surface RAGE, subsequently associated with increased expression of proinflammatory cytokines [18]. In addition, FLS are substantial sources of the osteoclastogenesis-promoting factor receptor activator of NFκB ligand (RANKL) and its soluble decoy receptor osteoprotegerin [19].

The influence of AGEs on FLS obtained from patients with OA has been, however, incompletely studied. We used AGE-BSA as a defined model system to study the potential effects on FLS. Our study demonstrates that AGE-BSA induce cell cycle arrest, proinflammatory changes and inhibition of osteoclastogenesis in cultured FLS obtained from OA patients. Thus, the effect of AGEs on FLS may likely contribute to the pathophysiology of OA.

## Materials and methods

### Reagents

The following reagents were used for cell isolation and culturing: DMEM (Gibco, Karlsruhe, Germany), RPMI 1640 (Promocell; Heidelberg, Germany), FCS (Lonza, Verviers, Belgium), gentamicin, Hepes (PAA Laboratories, Pasching, Austria), trypsin (Gibco, Karlsruhe, Germany), collagenase P (Roche Diagnostics, Mannheim, Germany) and Dynabeads CD14 (Invitrogen Dynal AS, Oslo, Norway). For the AGE-BSA preparation, fraction V, fatty acid-poor, endotoxin-free type of BSA was used (Calbiochem, La Jolla, CA, USA). For immunohistostaining and western blotting the following were used: primary antibodies anti-CD90 (AS02, Dianova, Hamburg, Germany); anti-CML (Roche Diagnostics, Penzberg, Germany); anti-imidazolone (kindly provided by Toshumitsu Niwa, Japan); anti-p27Kip1 (Cell Signaling Technology, Inc., Danvers, MA, USA); anti-RAGE (SP6366P, Acris Antibodies, Hiddenhausen, Germany); anti-NFκB p65, anti-IκB-αanti-pIκα (Santa Cruz Biotech, Santa Cruz, CA, USA); anti-β-actin and anti-vinculin (Sigma, St. Louis, MO, USA); horseradish peroxidase (HRP)-conjugated secondary antibodies (KPL, Gaithersburg, MD, USA); mouse and rabbit immunoglobulin (DakoCytomation, Glostrup, Denmark); Vectastain^® ^Elite ABC Kits (Vector Laboratories, Burlingame, CA, USA); complete Lysis-M buffer for protein extraction (Roche Diagnostics, Mannheim, Germany); BCA protein assay kit for quantification of total protein (Pierce, Rockford, IL, USA); Western Lightning Chemiluminescence Reagent Plus (Perkin Elmer LAS, Boston, MA, USA).

For cell proliferation and viability the following were used: bromodeoxyuridine (BrdU) and tetrazolium salt 3- [4,5-dimethylthiazol-2-yl]-2,5-diphenyl tetrazolium bromide (MTT) cell proliferation kits (Roche Diagnostics, Mannheim, Germany).

For cell cycle and cell death analysis the following were used: Annexin-V-FLUOS Staining Kit (Roche Diagnostics, Mannheim, Germany). For reverse transcriptase and real-time PCR the following were used: RNA lysis buffer, RNeasy Mini Kit, RNase-Free DNase Set (Qiagen, Hilden, Germany) for RNA extraction, Reverse Transcription System (Promega, Madison, WI, USA) for cDNA synthesis, FastStart DNA Masterplus SYBR Green I-Kit (Roche Diagnostics, Mannheim, Germany).

For cytokine measurements in culture supernatants the following were used: human TNF-α and IL-6 ELISA (R&D Systems, Minneapolis, MN, USA), osteoprotegerin and total soluble RANKL (sRANKL) ELISA (Immundiagnostik AG, Bensheim, Germany). For NFκB transactivation assay the following were used: pNFκB-Luc plasmid (Clontech Laboratories Inc., Mountain View, CA, USA), pSV-β-galactosidase plasmid (Promega, Madison, WI, USA), Lipofectamine Plus Reagent (Invitrogen, Carlsbad, CA, USA), Luciferase reporter assay system (Promega, Madison, WI, USA), Luminescent β-gal Reporter System 3 & Detection Kit II (Clontech, Mountain View, CA, USA). For electrophoretic mobility shift assay (EMSA) the following were used: NFκB consensus and mutant oligonucleotides, anti-NFκB p65(A)X (Santa Cruz Biotech, Santa Cruz, CA, USA), T4 Polynucleotide Kinase and Reaction Buffer (New England Biolabs Inc., Ipswich, MA, USA), [γ^32^P] ATP (Hartmann Analytic GmbH, Braunschweig, Germany), poly d(I-C) (Roche Diagnostics, Mannheim, Germany). For RAGE inhibition the following were used: anti-RAGE antibody (N-16; Santa Cruz Biotech, Santa Cruz, CA, USA).

### Patients

Synovial tissues were obtained at the time of knee replacements from 15 patients with OA (9 women, 6 men; 64.5 ± 9 years). Informed consent for the study was given by all patients and the study was approved by the local ethics committee.

**Figure 1:**
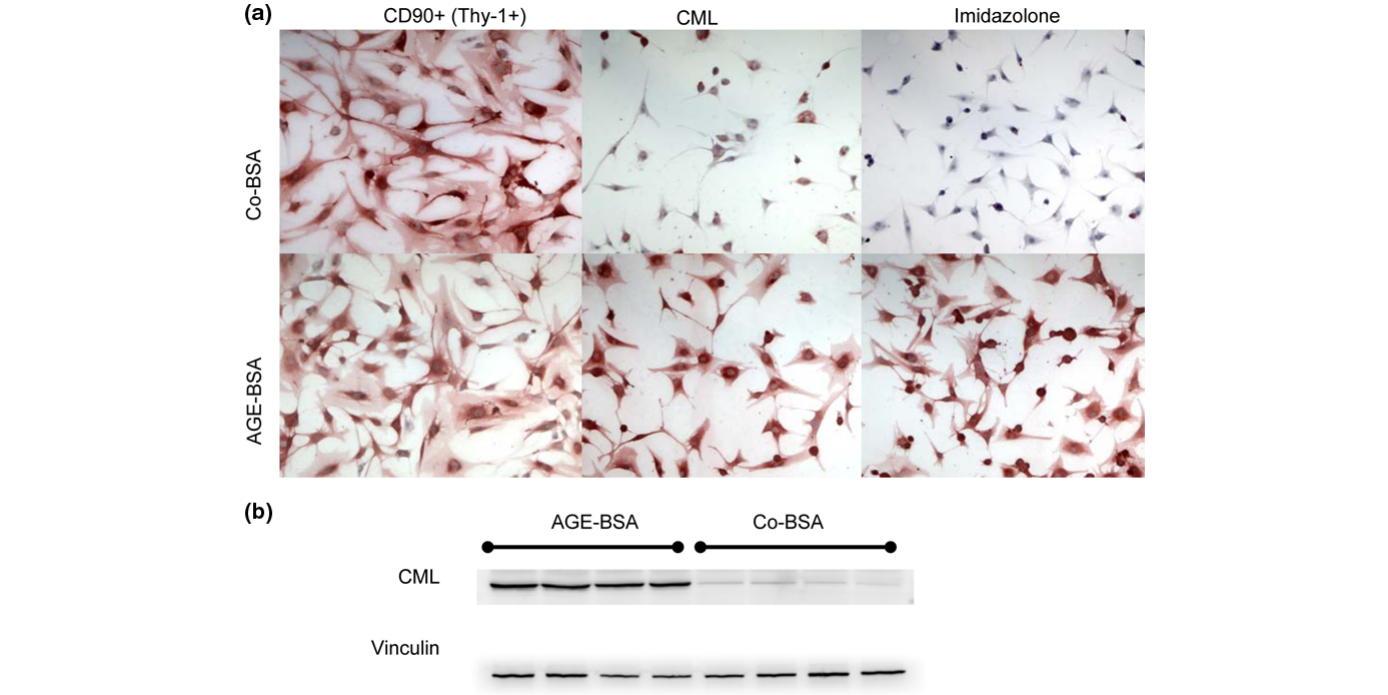
Characterisation of FLS and AGE uptake. **(a) **Immunohistochemical staining of fibroblast-like synovial cells (FLS) cultured from osteoarthritic synovial tissues. FLS were stimulated with control-BSA (Co-BSA) or advanced glycation end products-modified (AGE)-BSA (5 mg/ml) for 24 hours. FLS stained positive for the fibroblast marker CD90 and AGE-BSA incubation had no influence on CD90+ expression. The intensive intracellular staining for N^ε^-carboxymethyllysine (CML) and imidazolone in AGE-BSA treated cells in comparison with Co-BSA suggests active uptake of AGE. **(b) **Western blot for CML. FLS treated with AGE-BSA expressed more CML protein than cells incubated with Co-BSA.

The synovial samples were digested and subsequently cultured for seven days as described by Zimmermann and colleagues [20]. Briefly, synovial tissue was minced and digested at 37°C in PBS containing 0.1% trypsin for 30 minutes followed by 0.1% collagenase P in DMEM/10% FCS for two hours. After filtration through a sterile sieve (Sigma, St. Louis, MO, USA), cells were suspended in DMEM supplemented with 10% FCS, Hepes (25 mM) and gentamicin (100 μg/ml) and primary cultured for seven days at 37°C in a humidified atmosphere of 5% carbon dioxide (CO_2_) and 95% air. The media were changed on days one, three and five and non-adherent cells were removed. After one week, FLS were negatively isolated from trypsinised primary-culture synovial cells by depletion of monocytes/macrophages using Dynabeads M-450 anti-CD14 (Invitrogen Dynal, AS, Oslo, Norway) according to the manufacturer's protocol. FLS were then grown in DMEM supplemented as above. Only third to seventh passage cells were used for the experiments after the medium was replaced by RPMI 1640 (with 10% FCS and 100 μg/ml gentamicin). The large spindle-shaped cells of these passages were morphologically homogeneous and positive for CD90^+ ^(Thy-1^+^) as detected by immunohistochemical staining (Figure 1a)

In an additional experiment, human dermal fibroblasts were used to evaluate the specificity of the RANKL and osteoprotegerin expression data obtained in synovial FLS. Dermal fibroblasts were isolated from small skin pieces obtained from surgical resections performed for a variety of reasons (e.g. removal of subcutaneous lipoms). Histological evaluation showed normal skin structure. The specimens were minced, suspended in DMEM (with 10% FCS and 100 μg/ml gentamicin) and cultured at 37°C in 5% CO_2 _and 95% air. Outgrowing cells were isolated by trypsination two weeks later and expanded in DMEM with 10% FCS.

### Preparation of AGE-BSA

BSA was incubated under sterile conditions at 37°C for 50 days in PBS with and without the addition of glucose (90 mg/ml), then filtrated to remove unbound glucose and glucose degradation products (Millipore Labscale TFF System, Bedford, MA, USA), and lyophilised. After glycation, AGE-BSA was characterised by a 90-fold higher content of N^ε^-carboxymethyllysine (CML) than control-BSA (12.47 versus 0.14 nmol/mg protein in control-BSA (Co-BSA)) and a 10-fold higher pentosidine concentration (2.3 versus 22.8 pmol/mg protein in Co-BSA). CML was measured by an ELISA (Roche Diagnostics, Mannheim, Germany) and pentosidine by high performance liquid chromatography (Merck-Hitachi, Darmstadt, Germany) as previously described [21].

After optimising the dose and time course of AGE-BSA treatment all experiments were conducted in RPMI 1640 containing 0.1% FCS supplemented with 5 mg/ml AGE-BSA or 5 mg/ml Co-BSA (corresponding to 75 μmol/l). Cells were incubated for a period of up to seven days at 37°C in a humidified atmosphere of 5% CO_2 _and 95% air. For histochemical studies, cells were seeded in chamber slides (Nunc, Rochester, NY, USA) and treated as described before. AGE uptake of FLS was confirmed by immunohistochemical staining and western blot analysis for the detection of AGE-modified albumin.

### Immunohistochemical staining

For immunohistochemical staining, cells growing in chamber slides were fixed with 70% ethanol in a glycine buffer (150 mM glycine, 25 mM NaCl, 25 mM HCL) for 20 minutes at -20°C and then incubated with 3% hydrogen peroxide for 10 minutes at room temperature to block endogenous peroxidase. The following primary antibodies were used: anti-CD90, anti-CML and anti-imidazolone. Staining was performed using the Vectastain^® ^Elite ABC Kits and aminoethylcarbazole as a chromogen. Counterstaining was performed with Mayer's haematoxylin. For negative controls, primary antibodies were replaced by rabbit or mouse immunoglobulin in the same concentration as the primary antibody.

### Cell proliferation and viability tests

To evaluate the influence of AGE-BSA on the number of cultured cells, FLS were seeded in six-well plates. After 24 hours, the media were changed to RPMI 1640 containing either AGE-BSA or Co-BSA and incubated for a period of up to seven days. On days 1, 2, 3 and 7, cells were detached and counted (CASY Cell Counter, Innovatis, Reutlingen, Germany). To assess the FLS proliferation in response to Co-BSA or AGE-BSA treatment, BrdU incorporation was measured by a colorimetric assay as a parameter for DNA synthesis. For evaluation of cell viability and metabolic activity the MTT assay was used. The assay is based on the cleavage of tetrazolium salt (MTT) to coloured formazan by metabolic active cells, which occurs in viable cells only. FLS were grown in 96-well microtiter plates with 3000 cells per well in RPMI 1640 containing 10% FCS for 24 hours. Then, the media were changed into RPMI containing Co-BSA or AGE-BSA and incubated for another 16 hours. Subsequently, either BrdU or MTT labelling reagent was added for four hours. BrdU incorporation was measured at an absorbance of 450 nm and the solubilised formazan of the MTT assay at 570 nm. Each measurement was performed in six different FLS cell lines with eight per treatment group.

### Cell cycle analysis and evaluation of cell death

For cell cycle analysis FLS were harvested after 4, 8, 16, 24 and 48 hours after Co-BSA or AGE-BSA treatment, then stained with propidiumiodide and analysed by a flow cytometer (FACSCalibur, Becton Dickinson, Franklin Lake, NJ, USA). To investigate whether AGE-BSA induces early apoptosis and necrosis, FLS were stained with annexin-V-fluorescein and propidiumiodide simultaneously after one, two, three and seven days of Co-BSA or AGE-BSA incubation. Cell pellets were resuspended in Annexin FLUOS labelling solution (20 μl annexin-V-FLUOS^® ^labelling reagent and 20 μl propidiumiodide in 1 ml incubation buffer^®^) and incubated for 15 minutes at room temperature. Then, 0.5 ml incubation buffer^® ^was added per 10^6 ^cells. Analysis was performed using 488 nm excitation and a 515 nm band pass filter for fluorescein detection and a filter of more than 600 nm for propidiumiodide detection.

### Reverse transcriptase and real-time PCR

Total cellular RNA was extracted from treated FLS after direct lyses in the culture flasks using an RNA isolation kit according to the manufacturer's instructions. The standard protocol was supplemented by DNase digestion by using the corresponding RNase-Free DNase Set. RNA yield and purity was determined by measuring the absorbance at 260 and 280 nm. Complementary DNA (cDNA) was synthesised from 3 μg of total RNA with the Reverse Transcription System.

Real-time PCR was performed with the Realplex Mastercycler instrument (Eppendorf AG, Hamburg, Germany). For preparation of the Master Mix, the FastStart DNA Masterplus SYBR Green I-Kit was used. Together with the specific primers, the Master Mix was added to cDNA solutions. The cDNA samples were amplified according to the manufacturer's instructions. Non-template controls were included to ensure specificity. The sequences of the chosen primers and the cycler conditions are given in Table 1. The quantity of mRNA was calculated using the threshold cycle (Ct) value for amplification of each target gene and for human glyceraldehyde 3-phosphate dehydrogenase (GAPDH) as a reference gene. For comparing expression results between AGE-BSA and Co-BSA treatments, the 2^ΔΔCt ^formula was used for relative quantification [22].

**Table 1:** DNA sequences of the sense and antisense primers for real-time PCR analysis and cycler conditions

Gene	Accession number	Primer sequences	Annealing temperature (°C)	Number of cycles	Product size (bp)
GAPDH	[GenBank:J02642]	5'-CAATGACCCCTTCATTGACC-3' (sense) 5'-TGGACTCCACGACGTACTCA-3' (antisense)	59	30	197
IL-6	[GenBank:M14584]	5'-CTTTTGGAGTTTGAGGTATACCTAG-3' (sense) 5'-CGCAGAATGAGATGAGTTGTC-3' (antisense)	62	30	233
NFκB p65	[GenBank:NM_021975]	5'-AGTACCTGCCAGATACAGACGAT-3' (sense) 5'-GATGGTGCTCAGGGATGACGTA-3' (antisense)	62	30	215
Osteoprotegerin	[GenBank:U94332]	5'-TGCAGTACGTCAAGCAGGAG-3' (sense) 5'-CCCATCTGGACATCTTTTGC-3' (antisense)	53	30	175
p27^Kip1^	[GenBank:NM_004064]	5'-AGATGTCAAACGTGCGAGTG-3' (sense) 5'-TCTCTGCAGTGCTTCTCCAA-3' (antisense)	59	40	154
RAGE	[GenBank:AB036432]	5'-GGAAAGGAGACCAAGTCCAA-3' (sense) 5'-CATCCAAGTGCCAGCTAAGA-3' (antisense)	59	30	166
RANKL	[GenBank:AF019047]	5'-GCTTGAAGCTCAGCCTTTTG-3' (sense) 5'-CGAAAGCAAATGTTGGCATA-3' (antisense)	59	40	192
TNF-α	[GenBank:NM_000594]	5'-GGCAGTCAGATCATCTTCTCGAA-3' (sense) 5'-AAGAGGACCTGGGAGTAGATGA-3' (antisense)	62	40	195

GAPDH = glyceraldehyde 3-phosphate dehydrogenase;

NFκB = nuclear factor kappa B; RAGE = receptor for advanced glycation end products; RANKL = receptor activator of NFκB ligand.

### Western blot analysis

For western blot analysis, FLS stimulated with Co-BSA or AGE-BSA for 48 hours were lysed in complete Lysis-M buffer and the protein concentrations were determined using the BCA protein assay kit. In selected experiments, RAGE activation was blocked by addition of an anti-RAGE antibody to the cells 24 hours prior to AGE-BSA addition (N-16, 20 ng/ml). After incubating the protein extracts in sodium dodecyl sulphate (SDS) sample buffer at 100°C for five minutes, aliquots of 20 μg protein/lane were electrophoresed in a 12% acrylamide SDS-polyacrylamide gel. Proteins were transferred to a polyvinylidene fluoride membrane using a semidry transfer cell (Bio-Rad Laboratories, Hercules, CA, USA). Nonspecific binding sites were blocked for one hour with 5% BSA in Tris-buffered saline (Tris, pH 7.4) and 0.1% Tween-20 followed by overnight incubation at 4°C in primary antibodies to CML (polyclonal rabbit), p27^Kip1 ^(polyclonal rabbit), RAGE (polyclonal rabbit), NFκB p65 (monoclonal mouse), IκB-α, pIkB-α or to β-actin/vinculin (monoclonal mouse). The membrane was then washed four times for five minutes in Tris buffer containing 0.1% Tween-20, and incubated with the corresponding HRP-linked secondary antibody (KPL). Detection of peroxidase was performed with an enhanced chemiluminescent reagent (Western Lightning Chemiluminescence Reagent Plus). For imaging and digitisation the LAS-3000 imaging system (Fujifilm Life Science, Düsseldorf, Germany) was used. For quantification, the band densities were measured using the TotalLab TL120 Software (Nonlinear Dynamics, Newcastle, UK) and normalised for the respective densities of β-actin bands as loading controls.

### Measurement of TNF-α, IL-6, sRANKL and osteoprotegerin release

To assess the release of the proinflammatory cytokines IL-6 and TNF-α in FLS culture supernatants, concentrations were determined using cytokine-specific ELISA kits (R&D Systems, Minneapolis, MN, USA). For measurement the respective levels of the osteoclastogenesis-promoting factor sRANKL and its soluble decoy receptor osteoprotegerin, total sRANKL and osteoprotegerin ELISA kits (Immundiagnostik AG, Bensheim, Germany) were used. FLS were stimulated in six-well plates with Co-BSA or AGE-BSA for 48 hours. The conditioned media were harvested and stored at -80°C until the measurements were performed. Then, cells were detached and counted. The results were corrected by the numbers of FLS in the wells.

### NFκB transactivation assay

To test whether AGE-mediated NFκB activation leads to target gene binding and activation *in vivo*, FLS were transfected with the pNFκB-Luc reporter plasmid together with the pSV-β-galactosidase plasmid. The pNFκB plasmid contains four copies of the κ enhancer fused to the herpes simplex virus thymidine kinase promoter. Activation results in transcription of the luciferase gene. For transfection, FLS were seeded 24 hours before in six-well plates in RPMI/10%FCS. Then, cells were transfected with 4 μg pNFκB-Luc and the same amount pSV-β-galactosidase under serum-free conditions using Lipofectamine and Plus Reagent. After adding the transfection mix gently and drop wise, FLS were incubated over night. Subsequently, cells were stimulated with Co-BSA or AGE-BSA for 24 hours as appropriate. Luciferase activities were measured using a luciferase reporter assay system according to the manufacturer's protocol with a luminometer (LUMIstar OPTIMA, BMG Labtech GmbH, Offenburg, Germany). Luciferase activities were normalised to β-galactosidase activities determined by the corresponding Luminescent β-gal Reporter System 3 & Detection Kit II according to the manufacturer's instructions.

### Electrophoretic mobility shift assay for NFκB

FLS isolated from three different patients were grown on 100 mm dishes in RPMI with 10% FCS. To block RAGE activation, an anti-RAGE antibody was added to the cells 24 hours prior to AGE-BSA addition (20 ng/ml). Then cells were treated for one day with Co-BSA, AGE-BSA or AGE-BSA together with the RAGE-blocking antibody. In addition, TNF-α-stimulated FLS (10 ng/ml TNF-α for two hours) were used as a positive control for NFκB activation. EMSA of nuclear extracts was performed as previously described [23]. In detail, cells were washed with ice-cold PBS and lysed in 500 μl buffer (15 mM Tris-HCl, pH 7.9, 10 mM KCl, 2 mM MgCl_2_, 0.1 mM EGTA, 0.1 mM EDTA, 0.5 mM PMSF, 0.15% NP-40). Lysates were incubated on ice for 15 minutes, passed through a 26-gauge syringe and centrifuged at 5000 rpm for five minutes. The supernatant, containing the cytoplasmic proteins, was removed and 25 μl of nuclear extraction buffer (20 mM Tris-HCl pH 7.9, 0.4 M NaCl, 1 mM MgCl_2_, 5 mM EDTA, 0.5 mM DTT, 0.5 mM PMSF, 0.1% NP-40, 10% glycerol and an appropriate amount of protease cocktail inhibitors) were added to the pellet. Nuclei were incubated for 30 minutes on ice followed by centrifugation at 13,000 rpm for 30 minutes. The protein concentration was measured and the samples were aliquoted and stored at -80°C.

The double stranded NFκB consensus oligonucleotide was end-labelled using T4 polynucleotide kinase and [γ-^32^P] ATP (5000 Ci/mmol) followed by purification over a G-25 Sephadex column (GE Healthcare, Piscataway, NJ, USA). Binding reaction was carried out for 30 minutes at an ambient temperature and consisted of 3 μg of nuclear proteins, binding buffer (15 mM Tris-HCl, pH 7.9, 60 mM KCl, 1 mM MgCl_2_, 1 mM EDTA, 1 mM EGTA, 10% glycerol, 1 mM DTT), 2 μg of poly(dI-dC), 3 μg BSA and 40 fmol of labelled probe (450,000 cpm) in a total volume of 20 μl. In competition assays, the 100-fold molar excess of unlabelled oligonucleotides (NFκB consensus and mutant oligonucleotides, AP1 consensus oligonucleotide) were added 30 minutes prior to the addition of labelled probe.

The following sequences were used:

NFκB consensus           5'-AGTTGAGGGGACTTTCCCAGGC-3',

NFκB mutant                5'-AGTTGAGGCGACTTTCCCAGGC-3',

AP1 consensus                  5'-CGCTTGATGACTCAGCCGGAA-3'

The supershift antibody (400 ng) against NFκB p65 (Santa Cruz Biotech, Santa Cruz, CA, USA) was added to the reaction 30 minutes before the administration of the labelled probe.

The protein-DNA complexes were resolved on 6% polyacrylamide gel in Tris/Borate/EDTA (TBE)-buffer.

### Statistical analysis

All data are reported as means ± standard error of the mean. Statistical analysis was performed using SPSS 15 for Windows (SPSS, Chicago, IL, USA). Results were analysed with the Kruskal-Wallis test followed by the Mann-Whitney U-test. *P *values less than 0.05 were considered significant.

## Results

### Characterisation of FLS and AGE uptake

For characterisation of FLS cultured from synovial tissues, the presence of the fibroblastic marker protein CD90 (Thy-1) was demonstrated in Co-BSA as well as in AGE-BSA treated cells (Figure 1a). Cells grown in AGE-BSA and Co-BSA show the typical spindle-shaped form of fibroblasts (Figure 1a). Immunohistochemical staining for CML and imidazolone, representative members of the AGE family, demonstrates AGE-BSA uptake into the cytoplasm of the FLS (Figure 1a). This observation was confirmed by western blotting. AGE-BSA-treated FLS showed a strong accumulation of intracellular CML compared with cells receiving Co-BSA (Figure 1b).

### Cell proliferation, cell viability, cell cycle and evaluation of cell death

**Figure 2:**
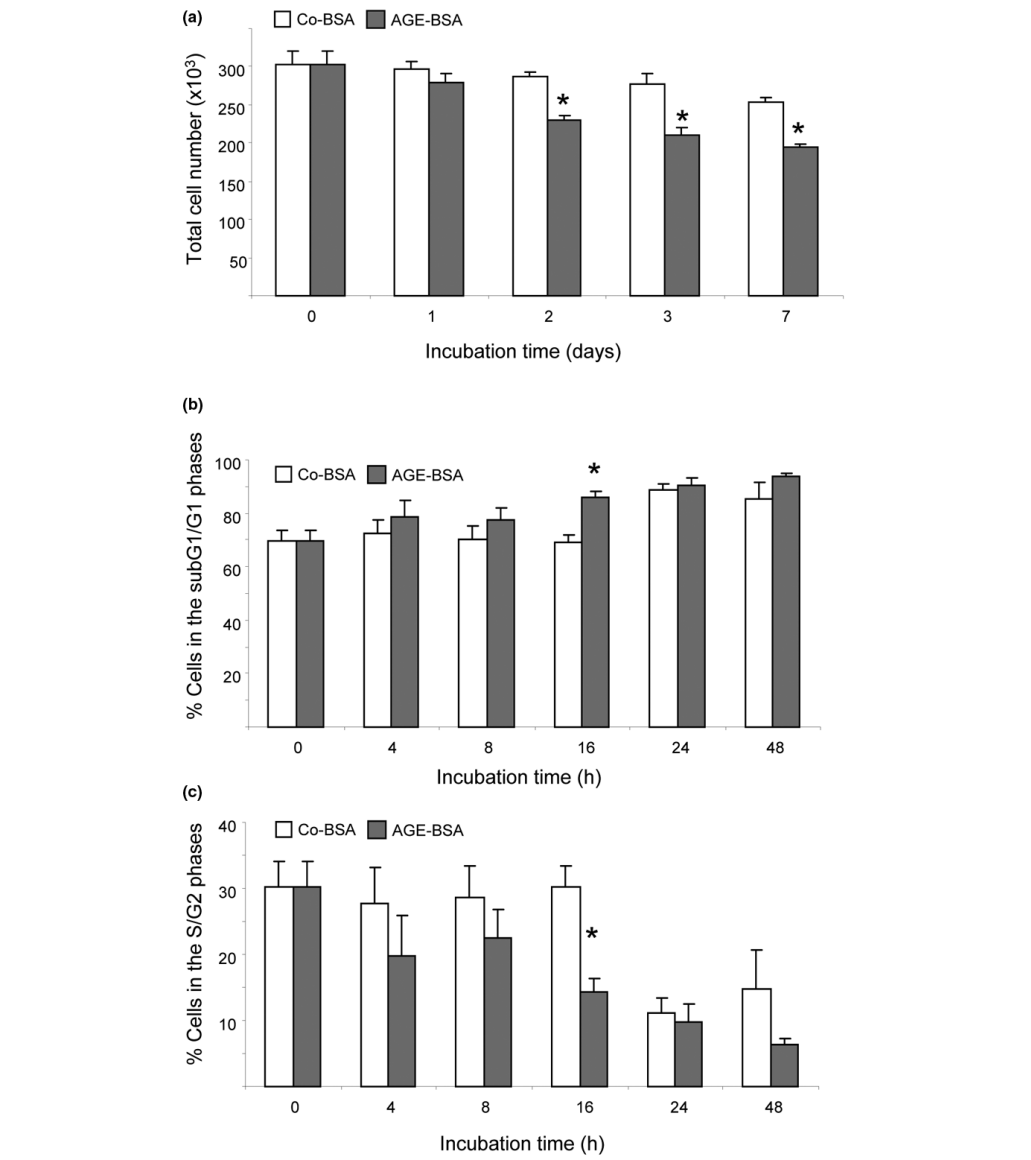
Cell cycle analysis of FLS. **(a) **Total cell number. Treatment of advanced glycation end products-modified (AGE)-BSA (5 mg/ml) significantly reduced total cell number after two to seven days in comparison with control-BSA (Co-BSA; *P < 0.001, n = 6). **(b) **Percentage of cells in the subG1 and G1 phases of the cell cycle. Incubation of fibroblast-like synovial cells (FLS) with AGE-BSA increased the percentage of cells in the subG1 and G1 phases. (*P < 0.01, n = 6). **(c) **Percentage of cells in the S and G2 phases. AGE-BSA significantly reduced after 16 hours the percentage of cells in the S and G2 phases (*P < 0.01, n = 6).

To evaluate whether AGE-BSA or Co-BSA treatment influences the survival of FLS, equal amounts of cells were cultured in media containing Co-BSA or AGE-BSA for a period of up to seven days. On days one, two, three and seven, FLS were detached and counted. As shown in Figure 2a, the total number of cells was significantly reduced from days two to seven by AGE-BSA treatment. For cell cycle analysis, FLS were harvested after 4, 8, 16, 24 and 48 hours of incubation in media containing either Co-BSA or AGE-BSA. After propidiumiodide staining, flowcytometric analysis was performed. In six independent experiments (FLS cell lines from six different patients), the total number of FLS in the subG_1_+G_1 _phase was significantly higher after 16 hours of AGE-BSA stimulation than for the respective Co-BSA treatment (Figure 2b). In contrast, the number of cells grown in the presence of AGE-BSA in the S+G_2 _phase was significantly lower compared with Co-BSA stimulated FLS (Figure 2c).

**Figure 3:**
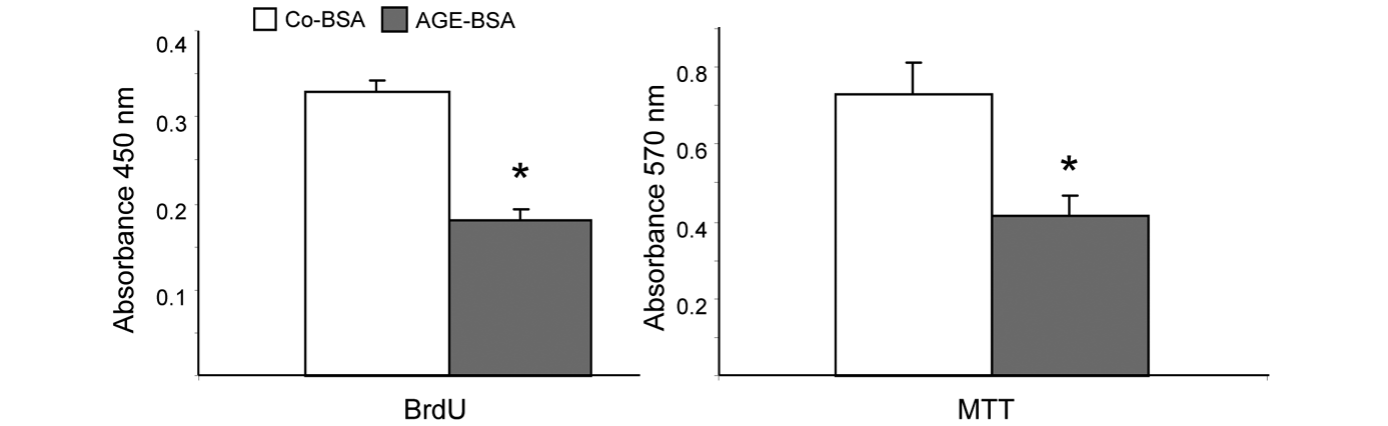
Cell proliferation and metabolic activity. Incubation of fibroblast-like synovial cells (FLS) for 16 hours with 5 mg/ml advanced glycation end products-modified (AGE)-BSA significantly reduced cell proliferation as measured by incorporation of bromodeoxyuridine (BrdU; *P *< 0.01, n = 6). Determination of metabolic activity in FLS with the MTT assay. AGE-BSA induced a significant decrease in metabolic activity of FLS (*P = 0.01, n = 6).

DNA synthesis was measured by BrdU incorporation and cell viability via metabolic activity by the MTT test. Figure 3 clearly demonstrates that AGE-BSA treatment in comparison with Co-BSA significantly reduces DNA synthesis as well as metabolic activity reflecting decreased proliferation and viability.

**Figure 4:**
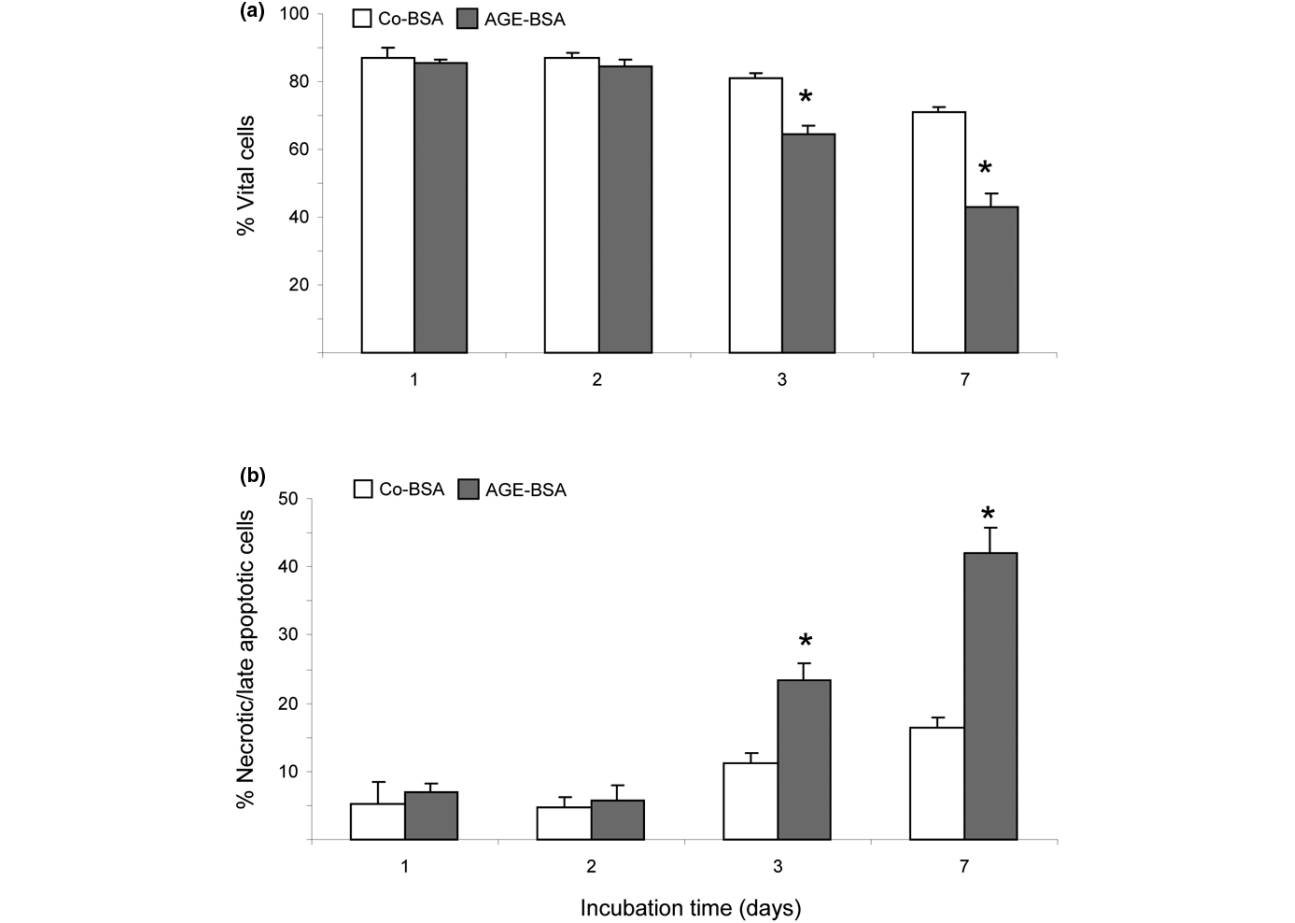
Quantification of cell death. **(a) **Percentage of vital cells as measured by FACS analysis (annexin-V and propidiumiodide negative). Incubation of cells with advanced glycation end products-modified (AGE)-BSA significantly reduced the number of vital cells from day three (*P < 0.05, n = 4). **(b) **Percentage of necrotic and late apoptotic cells (annexin-V and propidiumiodide positive) increased three to seven days during treatment with 5 mg/ml AGE-BSA (*P < 0.05, n = 4).

To test whether AGE-BSA induces apoptotic or necrotic cell death, FLS were analysed after annexin-V-fluorescein staining by flow cytometric analysis. A significant decrease of vital cells (annexin-V and propidiumiodide negative) was accompanied by a significant increase of necrotic and late apoptotic cells (annexin-V and propidiumiodide positive) after three days of AGE-BSA incubation (Figure 4). An increase in AGE-induced early apoptotic cells (annexin-V positive and propidiumiodide negative) could not be detected.

### p27^Kip1 ^expression

**Figure 5:**
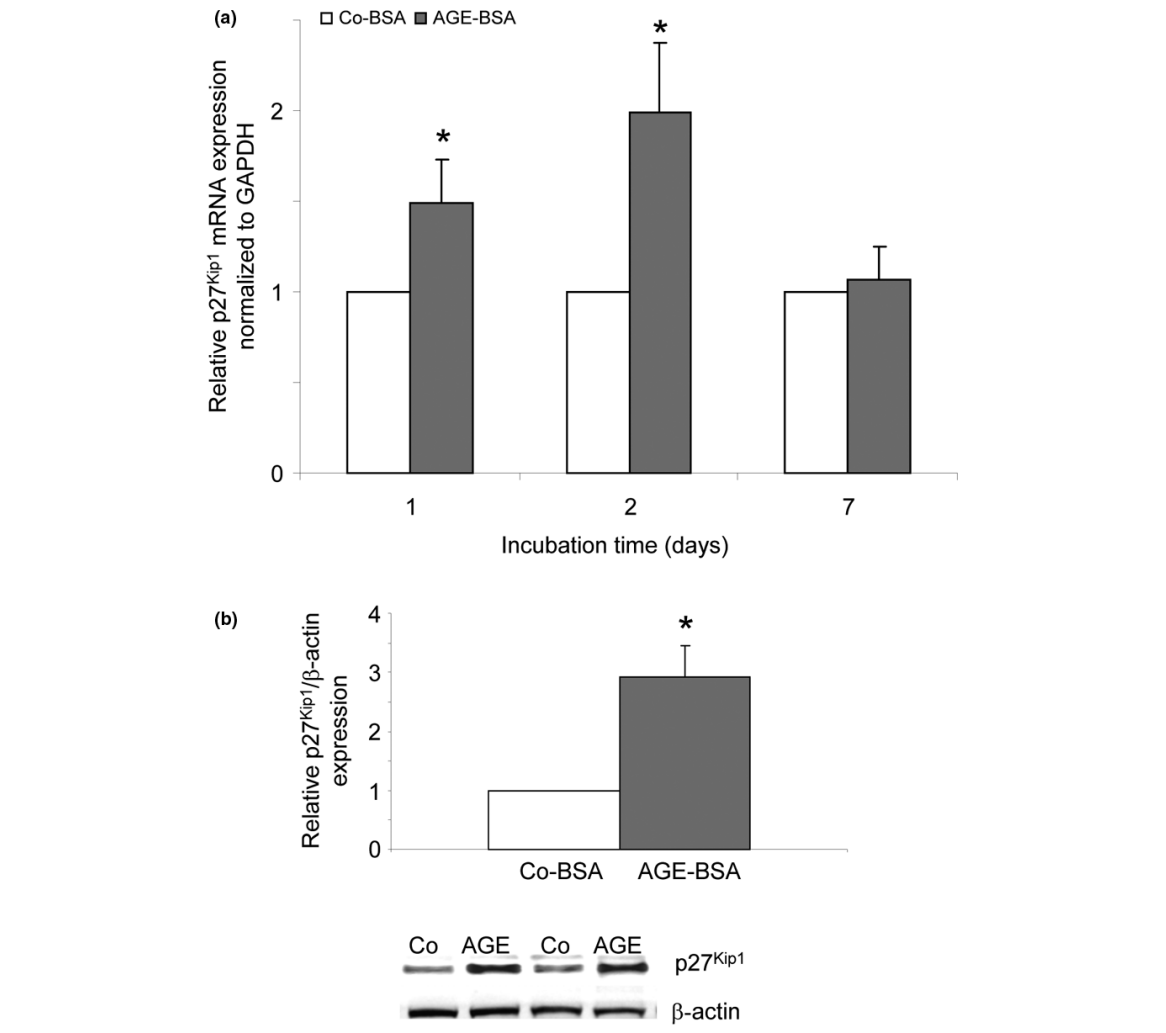
p27^Kip1 ^expression in FLS. **(a) **p27^Kip1 ^mRNA expression was significantly higher after one and two days of treatment with advanced glycation end products-modified (AGE)-BSA (*P < 0.01, n = 10). **(b) **Western blot for p27^Kip1 ^protein expression. 5 mg/ml AGE-BSA for 48 hours significantly increased p27^Kip1 ^protein expression (*P < 0.01, n = 6). Two representative western blots are shown. FLS = fibroblast-like synovial cells; GAPDH = glyceraldehyde 3-phosphate dehydrogenase.

To evaluate whether the cell cycle inhibitor protein p27^Kip1 ^is involved in the observed arrest of FLS in the subG1+G1 phase, cells were treated for up to seven days with either Co-BSA or AGE-BSA. p27^Kip1 ^mRNA expression of 10 individual cell lines was measured by real-time PCR. For western blot analysis, protein lysates after two days of treatment were used. The mRNA expression was found to be significantly upregulated after one and two days of AGE-BSA stimulation (Figure 5a) confirmed by a significantly higher protein expression at day 2 (Figure 5b).

**Figure 6:**
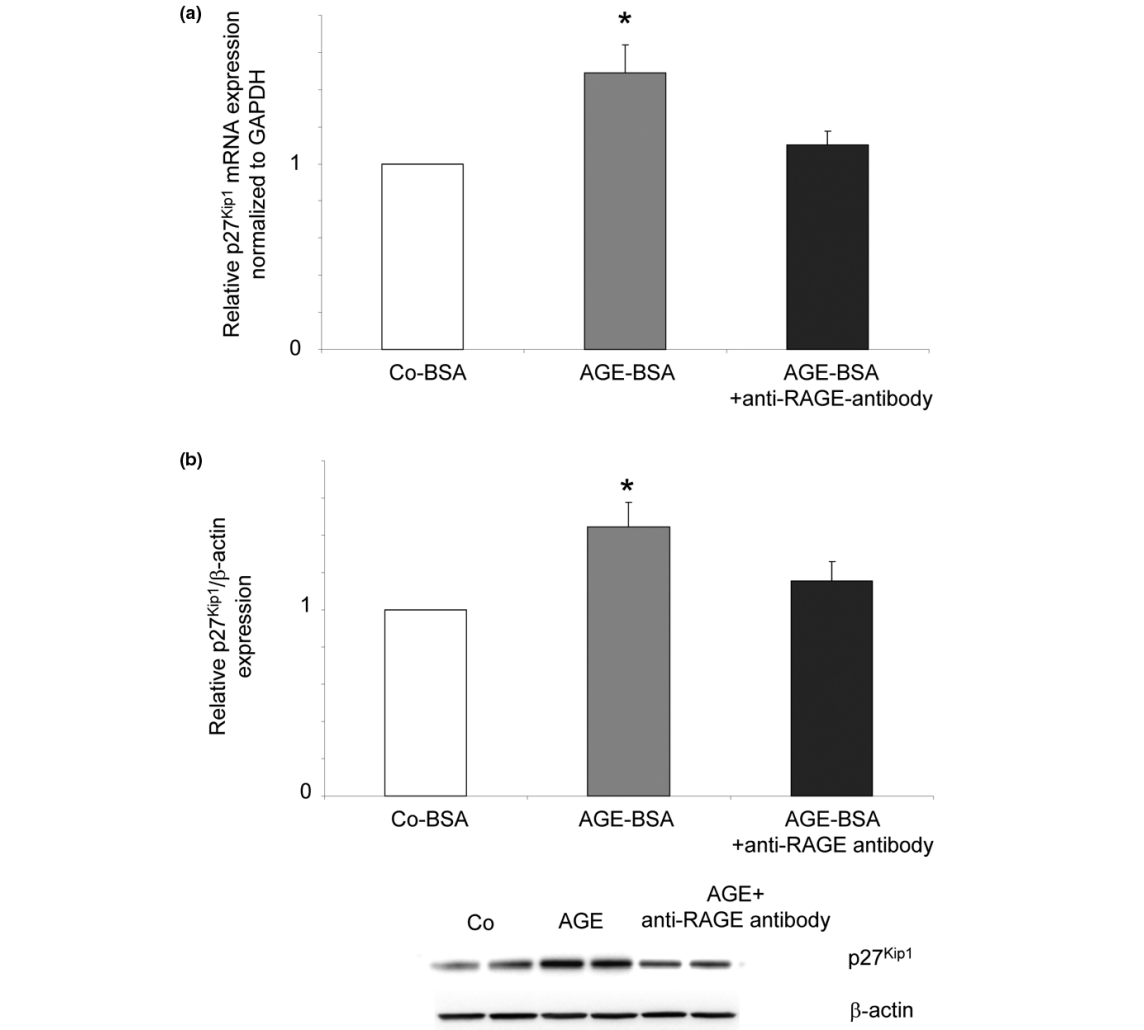
Inhibition of p27^Kip1 ^expression by an anti-RAGE antibody. Fibroblast-like synovial cells (FLS) were incubated for 24 hours with either control-BSA (Co-BSA), advanced glycation end products-modified (AGE)-BSA or AGE-BSA together with the anti- receptor for AGEs (RAGE) antibody **(a) **The significant increase of p27^Kip1 ^mRNA expression was inhibited when RAGE was blocked (AGE-BSA + anti-RAGE antibody versus AGE-BSA: *P *< 0.03, n = 5). **(b) **Western blot for p27^Kip1 ^protein expression. The AGE-BSA-induced increase in p27^Kip1 ^protein expression was abolished in the presence of the RAGE-blocking antibody (AGE-BSA + anti-RAGE antibody versus AGE-BSA: *P *< 0.05, n = 5). A representative western blot is shown underneath. GAPDH = glyceraldehyde 3-phosphate dehydrogenase.

To test whether the p27^Kip1 ^upregulation was mediated by RAGE, a neutralising antibody against RAGE was added to the cells 24 hours prior to AGE-BSA addition (N-16, 20 ng/ml). FLS of five different patients were incubated for one day with either Co-BSA, AGE-BSA or AGE-BSA together with the anti-RAGE antibody. As shown in Figures 6a and 6b, the AGE-BSA-induced increase in p27^Kip1 ^mRNA and protein expression was abolished in the presence of the antibody. This indicates that the observed p27^Kip1 ^induction was mediated by AGE-RAGE interactions.

### RAGE expression

**Figure 7:**
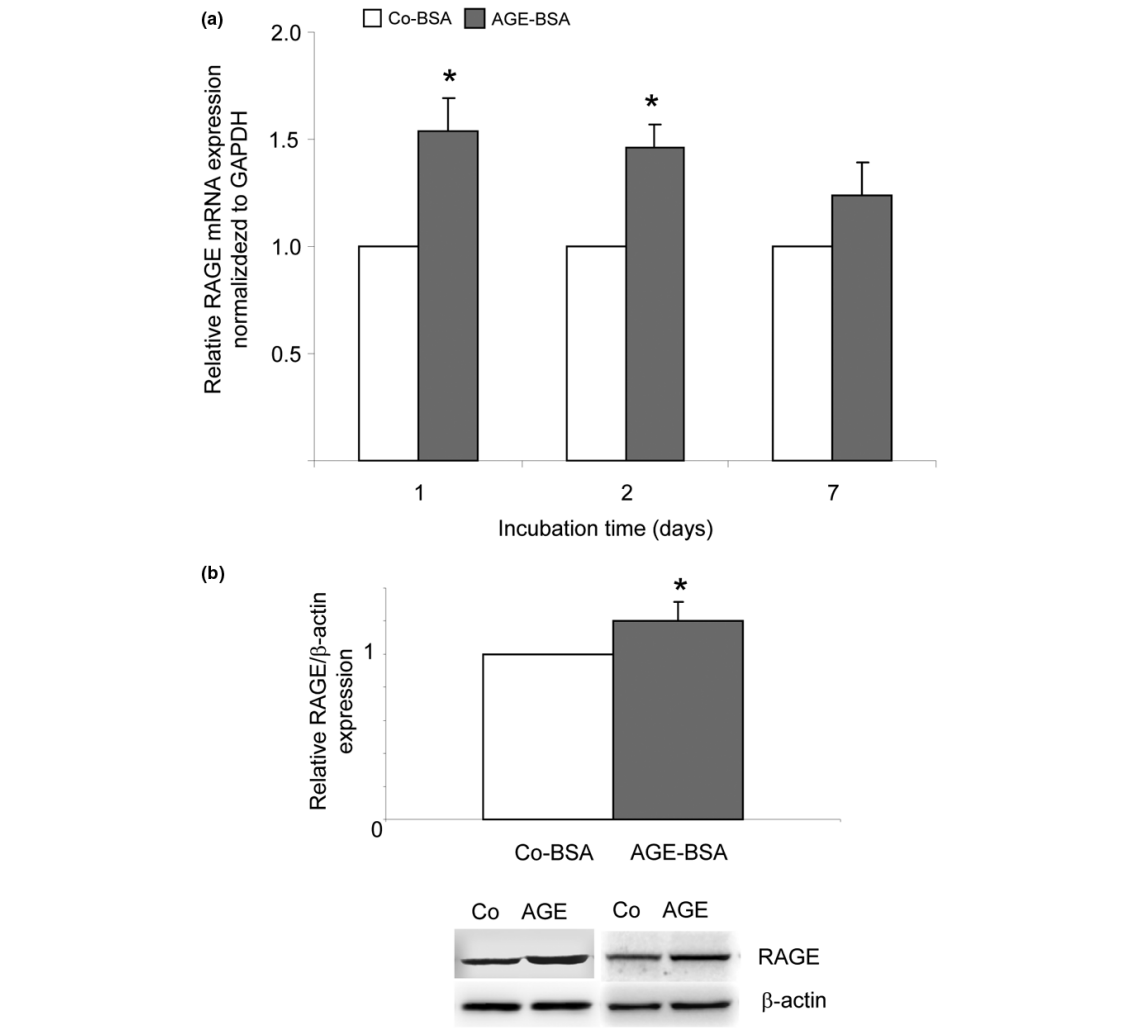
RAGE expression in FLS. **(a) **Real-time RT-PCR for the determination of receptor for AGEs (RAGE) mRNA expression. Incubation of cells with advanced glycation end products-modified (AGE)-BSA after one day induced the upregulation of RAGE mRNA (*P < 0.001, n = 15). **(b) **RAGE protein expression as determined by western blots. 5 mg/ml AGE-BSA for 48 hours significantly induced RAGE protein expression (*P < 0.001, n = 8). Examples of two independent western blots are shown. FLS = fibroblast-like synovial cells; GAPDH = glyceraldehyde 3-phosphate dehydrogenase.

Binding of AGEs to RAGE contributes to the activation of redox-sensitive transcription factors such as NFκB and subsequently induced expression of proinflammatory cytokines such as TNF-α and IL-6 [24]. To investigate whether the RAGE expression of FLS was influenced by AGE-BSA treatment, cells were incubated over seven days with either Co-BSA or AGE-BSA. RAGE mRNA expression of 15 individual cell lines was measured after one, two and seven days of incubation. For western blot analysis, cells of eight different cell lines were harvested and lysed after two days of treatment. As shown in Figure 7a, in comparison to Co-BSA the RAGE mRNA expression of AGE-BSA-stimulated cells was significantly upregulated after one and two days. For day two, the real-time PCR result was confirmed by western blot analysis also demonstrating a significant increased RAGE protein expression (Figure 7b).

### NFκB p65 expression and activation

**Figure 8:**
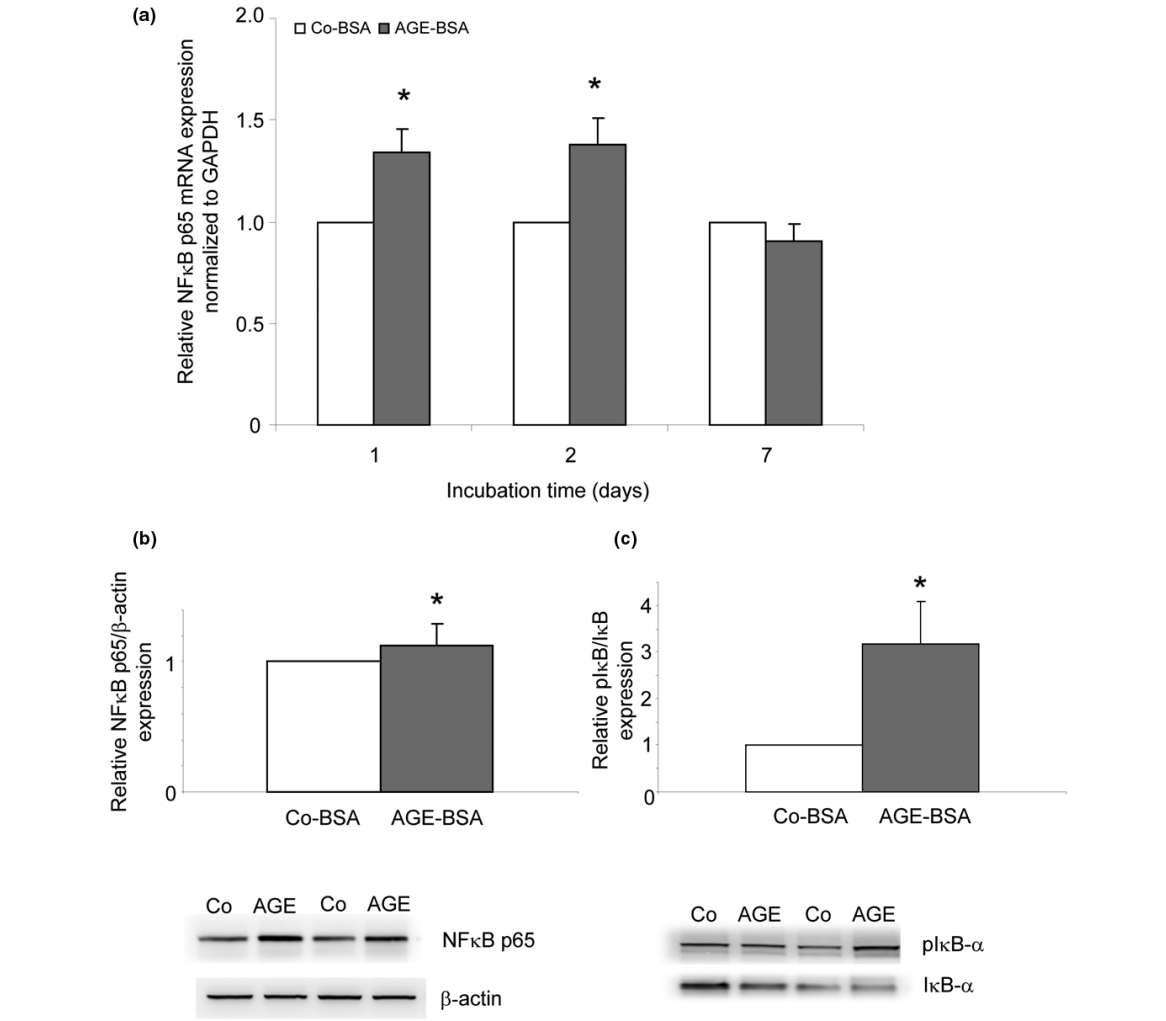
NFκB activation. **(a) **mRNA expression for the p65 nuclear factor kappa B (NFκB) subunit (real-time RT-PCR). Advanced glycation end products-modified (AGE)-BSA significantly increased p65 transcripts after one and two days (*P < 0.001, n = 15). **(b) **Western blot for p65 NFκB subunit protein expression. AGE-BSA in comparison with control-BSA (Co-BSA) significantly stimulated p65 protein expression two days after stimulation (*P < 0.001, n = 8). **(c) **Western blot for IκB-α and phosphorylated IκB-α (pIκB-α). Incubation of fibroblast-like synovial cells (FLS) for two days with 5 mg/ml AGE-BSA increased the amount of pIκB-α and decreased the amount of IκB-α resulting in a significantly higher pIκB-α/IκB-α ratio (*P < 0.001, n = 8). GAPDH = glyceraldehyde 3-phosphate dehydrogenase.

mRNA and protein expression of the NFκB subunit p65 was measured. The mRNA expression of p65 was significantly upregulated after one and two days of AGE-BSA incubation (Figure 8a) resulting in a significantly higher protein expression as detected by western blot analysis (Figure 8b). In resting cells, NFκB is localised in the cytoplasm in its inactive form bound to the inhibitor molecule IκB-α. Upon activation, IκB-α is rapidly phosphorylated and degraded resulting in the release and translocation of NFκB into the nucleus [12]. To study the effects of AGE-BSA on NFκB activation, western blotting of IκB-α and pIκB-α was performed. The IκBα protein expression after two days was lower in AGE-BSA-treated cells than in cells incubated with Co-BSA resulting in a significantly higher pIκBα/IκBα ratio (Figure 8c).

**Figure 9:**
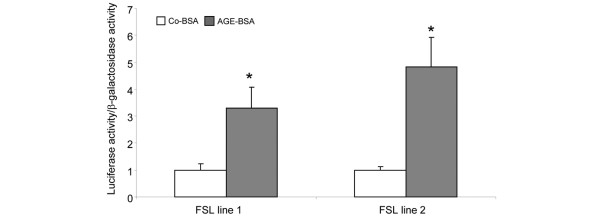
*In vivo *transactivation of NFκB. A luciferase reporter plasmid containing consensus binding sites for nuclear factor kappa B (NFκB) was transfected in two different fibroblast-like synovial cell (FLS) lines. Advanced glycation end products-modified (AGE)-BSA significantly enhanced NFκB transactivation compared with control-BSA (Co-BSA; *P < 0.05, n = 6).

To confirm the AGE-BSA-mediated NFκB activation *in vivo*, a reporter plasmid containing four tandem copies of the κ enhancer was transfected into two FLS cell lines. After transfection, FLS were incubated for 24 hours with either Co-BSA or AGE-BSA, then harvested and prepared for luciferase assay. As shown in Figure 9, the luciferase activity normalised to β-galactose activity was significantly higher in both investigated cell lines in AGE-BSA-treated cells as compared with Co-BSA.

**Figure 10:**
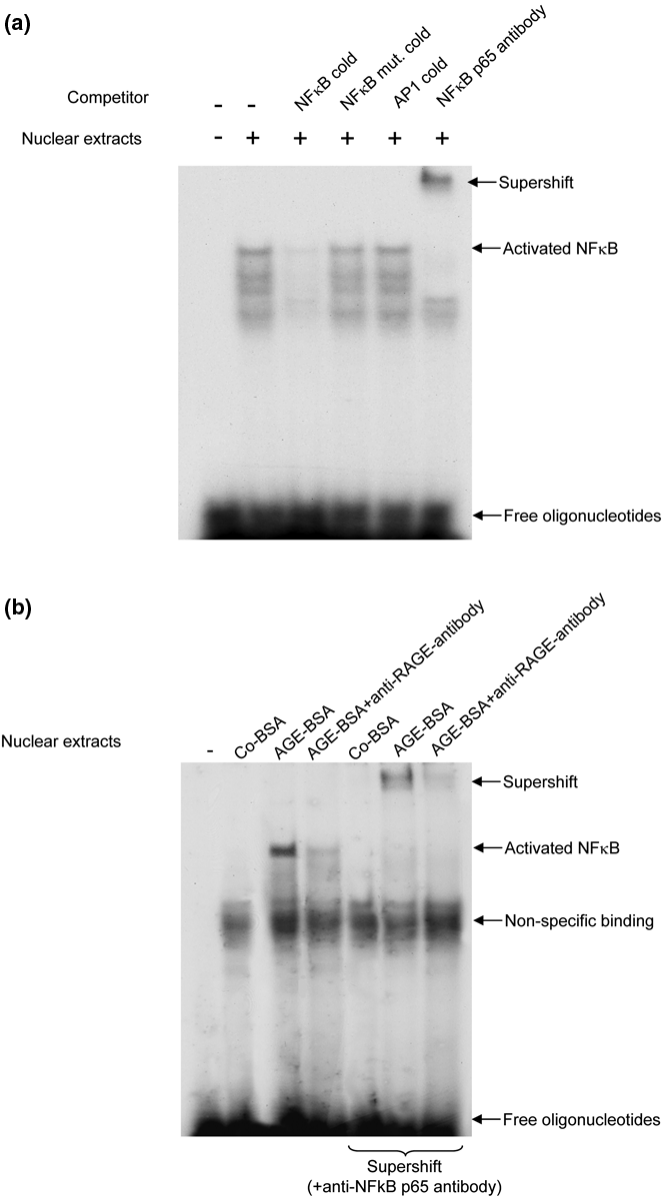
EMSA for NFκB. **(a) **A control experiment was performed to demonstrate the specificity of the assay. Aliquots of the nuclear extracts of TNF-α-activated fibroblast-like synovial cells (FLS; 10 ng/ml TNF-α for two hours) were incubated without (-) or with the indicated unlabelled oligonucleotides in the competition assays. The DNA-binding was reduced in the presence of cold nuclear factor kappa B (NFκB) probe, but not with NFκB mutant or AP1 oligonucleotides. An anti-NFκB p65 antibody induced a supershift. Data are representative of three separate experiments. **(b) **Advanced glycation end products-modified (AGE)-BSA, but not control-BSA (Co-BSA) treatment results in NFκB activation and the formation of NFκB-DNA complexes. Nuclear proteins isolated from AGE-BSA treated FLS in the presence of a receptor for AGEs (RAGE)-neutralising antibody showed a weaker NFκB binding in comparison with AGE-BSA stimulation alone. This demonstrates that the AGE-induced NFκB activation was caused by AGE-RAGE interactions. The specificity of NFκB binding was confirmed by supershifts using the NFκB p65 antibody. The shown electrophoretic mobility shift assay (EMSA) is representative of two independent experiments with similar results.

This finding is supported by EMSA experiments investigating the NFκB activation of Co-BSA and AGE-BSA-stimulated FLS *in vitro*. First, a control experiment was performed to demonstrate the specificity of the assay (Figure 10a). Aliquots of the nuclear extracts of TNF-α-activated FLS were incubated without (-) or with the indicated unlabelled oligonucleotides in the competition assays. The DNA-binding was reduced in the presence of cold NFκB probe, but not with NFκB mutant or AP1 oligonucleotides. Finally, supershift experiments in the presence of an anti-NFkB p65 antibody clearly confirmed the specificity of the binding reaction.

As shown in Figure 10b, AGE-BSA, but not Co-BSA treatment, results in NFκB activation and the formation of NFκB-DNA complexes. When RAGE activation was blocked by the anti-RAGE antibody, AGE-BSA-treated FLS showed only marginally NFκB binding as reflected by the lower intense band in comparison to AGE-BSA stimulation alone. This result clearly demonstrates that the AGE-induced NFκB activation in FLS was caused by AGE-RAGE interactions. The specificity of NFκB binding in these experiments was confirmed by supershifts using the NFκB p65 antibody and also the supershifted band was reduced in the presence of the anti-RAGE antibody.

### Expression of the proinflammatory cytokines TNF-α and IL-6

**Figure 11:**
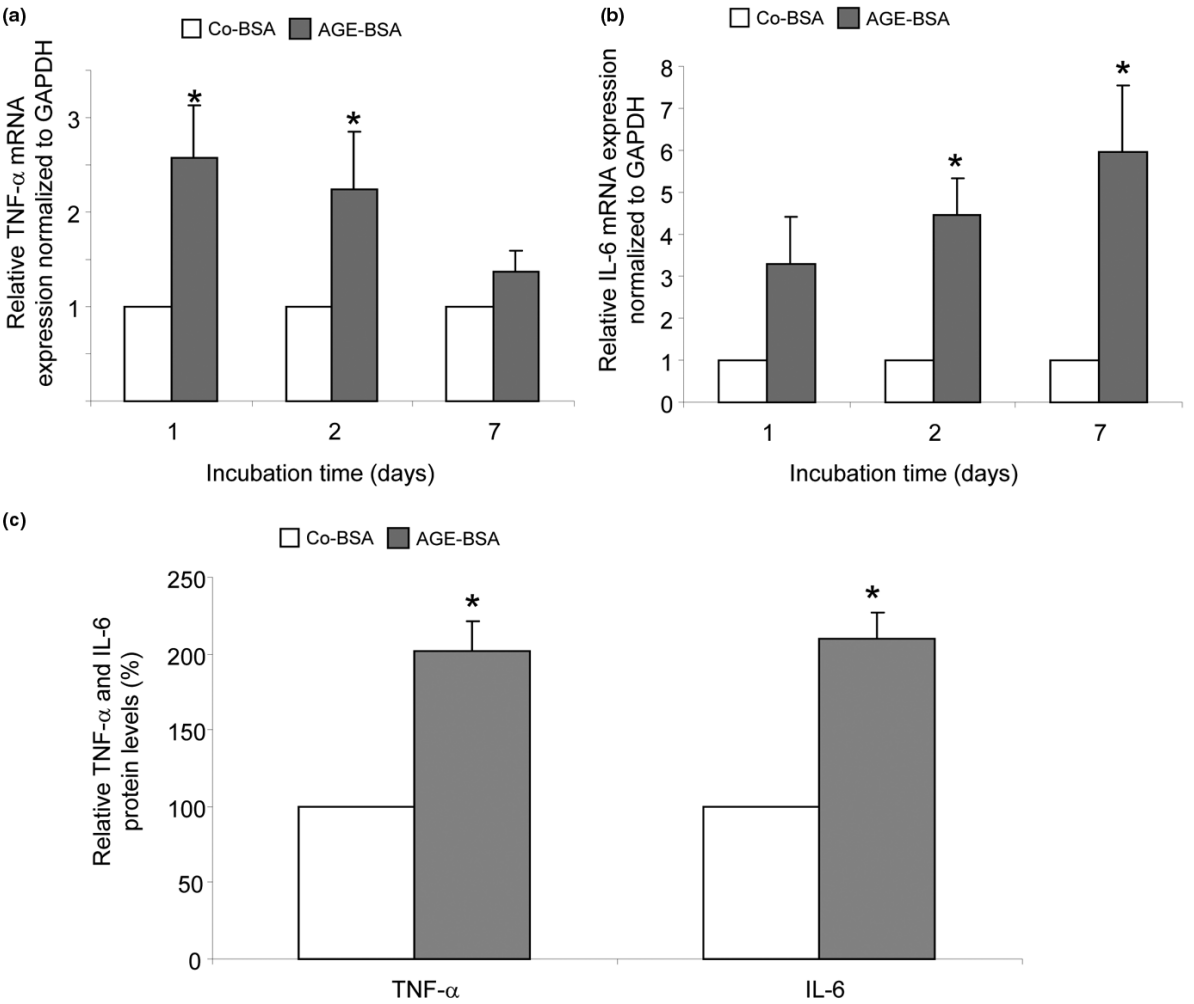
Stimulation of TNF-α and Il-6 by AGE-BSA. **(a) **Transcripts for TNF-α were significantly increased after one and two days (*P < 0.01, n = 15). **(b) **IL-6 mRNA expression was significantly increased two to seven days after advanced glycation end products-modified (AGE)-BSA treatment (*P < 0.001, n = 15). **(c) **Stimulation of fibroblast-like synovial cells (FLS) with AGE-BSA released significant more TNF-α as well as IL-6 into the culture supernatants as treatment with control-BSA (Co-BSA). Concentrations of TNF-α and IL-6 in the supernatant were normalised to cell number (*P < 0.01, n = 6). GAPDH = glyceraldehyde 3-phosphate dehydrogenase.

Because NFκB activates transcription of proinflammatory cytokines such as TNF-α and IL-6, we next tested the expression of these proinflammatory cytokines. The mRNA expression for TNF-α (Figure 11a) and IL-6 (Figure 11b) was significantly upregulated after two to seven days of AGE-BSA treatment compared with Co-BSA incubation.

To investigate the protein release of both cytokines into the culture supernatants, ELISA assays were used. The measured levels were normalised to the number of cells in the wells. Supernatants from six individual cell lines were examined. Significantly increased TNF-α and IL-6 levels were found in the media of AGE-BSA-stimulated FLS compared with Co-BSA treatment (AGE-BSA versus Co-BSA stimulation: TNF-α 12.7 ± 2.0 versus 6.2 ± 0.5 pg/10^5 ^cells; IL-6 173 ± 38 versus 81 ± 14 pg/10^5 ^cells). The relative increase of TNF-α and IL-6 release in the media of AGE-BSA treated cells in comparison to Co-BSA is shown in Figure 11c.

### Expression of RANKL and osteoprotegerin

**Figure 12:**
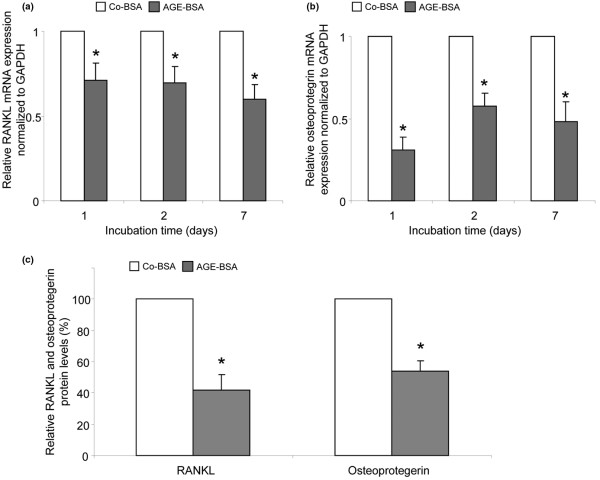
Reduction of RANKL and osteoprotegerin expression in FLS after incubation with AGE-BSA. **(a) **Receptor activator of nuclear factor kappa B ligand (RANKL) mRNA expression was significantly lower after treatment with advanced glycation end products-modified (AGE)-BSA (*P < 0.001, n = 15). **(b) **In addition, osteoprotegerin mRNA expression was significantly reduced by AGE-BSA (*P < 0.001, n = 8). **(c) **AGE-BSA reduced the release of RANKL and osteoprotegerin proteins into fibroblast-like synovial cells (FLS) supernatants compared with control-BSA (Co-BSA; *P < 0.01, n = 6). GAPDH = glyceraldehyde 3-phosphate dehydrogenase.

Synovial fibroblasts participate in osteoclastogenesis by expressing the osteoclastogenesis-promoting factor RANKL and its soluble decoy receptor osteoprotegerin. They are substantial sources of RANKL and osteoprotegerin *in vivo *[19]. Consequently, we also investigated whether the expression of RANKL and osteoprotegerin in FLS is influenced by AGE-BSA. RANKL mRNA expression was measured after one, two and seven days of stimulation in 15 and osteoprotegerin in 8 different cell lines. In opposite to RAGE, NFκB and the proinflammatory cytokines TNF-α and IL-6, the mRNA levels of RANKL and osteoprotegerin were significantly lower in AGE-BSA-treated FLS compared with cells receiving Co-BSA (Figures 12a, b). The same was found for RANKL and osteoprotegerin proteins released into the culture supernatant after AGE-BSA treatment for two days (AGE-BSA versus Co-BSA stimulation: sRANKL 73 ± 29 versus 192 ± 104 pg/10^5 ^cells; osteoprotegerin 0.7 ± 0.2 versus 1.2 ± 0.3 pg/10^5 ^cells). The relative changes are shown in Figure 12c.

**Figure 13:**
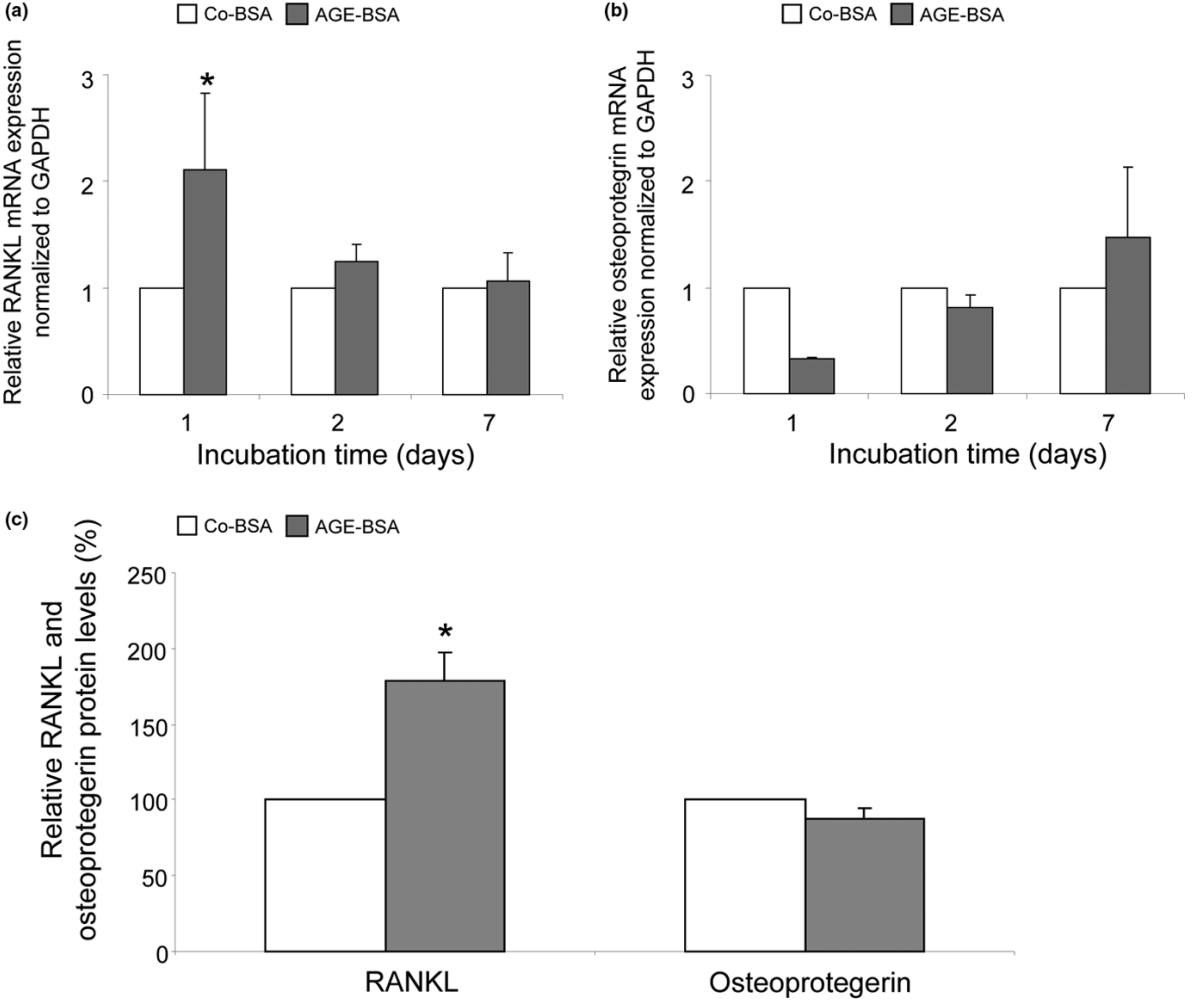
RANKL and osteoprotegerin expression of dermal fibroblasts after incubation with AGE-BSA. **(a and c) **Contrary to fibroblast-like synovial cells (FLS), advanced glycation end products-modified (AGE)-BSA significantly upregulated the receptor activator of nuclear factor kappa B ligand (RANKL) mRNA and protein expression of dermal fibroblasts. (*P < 0.05, n = 3). **(b and c) **The respective mRNA osteoprotegerin expression was significantly reduced after one day of AGE-BSA (*P < 0.05, n = 3). However, the osteoprotegerin protein level was reduced only marginally by AGE-BSA in dermal fibroblasts. GAPDH = glyceraldehyde 3-phosphate dehydrogenase.

To evaluate whether these results are specific for osteoarthritic FLS, a similar experiment was performed. In contrast to the FLS cells, AGE-BSA upregulated the RANKL mRNA and protein expression of dermal fibroblasts (Figures 13a and 13c). As shown in Figures 13b and 13c, the respective osteoprotegerin expression levels were reduced after one and two days of AGE-BSA stimulation and thus comparable with those of FLS. From these findings it can be concluded that the AGE-induced downregulation of RANKL expression is specific for osteoarthritic FLS probably reflecting a diminished osteoclastogenic capacity.

## Discussion

The molecular and cellular mechanisms of OA are still not completely understood. However, the importance of synovitis in the pathophysiology of OA is increasingly recognised. Chronic inflammatory changes with the production of proinflammatory cytokines are described as a feature of synovial membranes from patients with early OA [25]. Compared with late OA, increased mononuclear cell infiltration and overexpression of proinflammatory mediators (such as TNF-α and NFκB) were seen in synovium during the early phase of the disease, which may reflect increased activation of interrelated pathophysiological pathways that contribute to progressive joint damage [26]. FLS are involved in osteoarthritic synovial inflammation. FLS obtained from OA patients revealed a steady-state expression of TNF-α and IL-6 in culture [6]. FLS express RANKL and osteoprotegerin and therefore have the potential to participate in osteoclastogenesis.

AGEs extensively accumulate in cartilage collagen with age [27]. In a canine model of OA it has been shown that higher cartilage AGE levels increase the severity of OA and thus provide evidence for a molecular mechanism by which ageing predisposes to the development of OA [28]. Cartilage degradation induces the release of AGE-modified molecules. Access of these molecules to the synovium may affect synovial cells by direct AGE-cell contact. Studying molecular mechanisms of AGEs *in vivo *is difficult and therefore we relied on cell cultures of human FLS as an accepted *in vitro *model. We investigated the capacity of AGE-BSA to modulate cell cycle, proinflammatory changes and osteoclastogenesis in cultured FLS obtained from patients with OA.

We used AGE-BSA as a model system with standardised concentrations of specific AGEs such as CML and pentosidine. AGE-BSA is actively incorporated into cultured FLS and induced to cell cycle arrest, decreased cell proliferation and viability, but also increased expression of RAGE, NFκB p65 and of the proinflammatory cytokines TNF-α and IL-6. In contrast, AGE-BSA decreased expression of RANKL and osteoprotegerin in FLS. Although it is possible that FLS *in vivo *may be exposed to AGE-modified albumin under pathophysiological conditions, it is more likely that AGE-modified collagens may be potential culprits in OA. However, it has been difficult to reliable glycate very large proteins such as collagens and great variations from batch to batch of CML and pentosidine content were noted. Therefore, we relied on AGE-BSA for these *in vitro *studies to define basic molecular mechanisms of how AGEs may influence FLS.

Decreased proliferation and viability induced by AGEs is reported for a variety of cells including cardiac and skin fibroblasts, tubular epithelial cells and podocytes [29-32]. For podocytes, it was shown that the cell cycle inhibitor protein p27^Kip1 ^is involved in AGE-induced cell cycle arrest. Thus, we measured the p27^Kip1 ^mRNA and protein expression in AGE-stimulated FLS and found significantly increased levels indicating the important role of p27^Kip1 ^expression in AGE-BSA-mediated cell cycle arrest in FLS. The observation that AGE-BSA induces necrosis and late apoptosis but not early apoptosis in FLS agrees with these findings because cells arrested in the cell cycle become sensitive for necrosis. The release of potential proinflammatory material from necrotic FLS may participate in inflammatory responses. In contrast, AGE-mediated apoptosis has been recently described for fibroblasts and osteoblasts [33,34]. In these studies, CML-modified collagen was used and not AGE-BSA, which is modified with a variety of AGE structures including CML. This may explain the different AGE-induced mechanisms.

Our data clearly demonstrate that AGEs increase the inflammatory potential of FLS by activating RAGE and NFκB, which leads to increased expression of proinflammatory cytokines TNF-α and IL-6. Our experiments using the anti-RAGE antibody clear showed that this receptor is necessary for AGE-BSA mediated p27^Kip1 ^induction as well as NFκB activation.

These data together with the AGE-induced upregulation of metalloproteinases reported by Steenvoorden and colleagues may be evidence for the capacity of AGEs to modulate FLS towards inflammation and cartilage degradation and amplify OA [17]. As the RAGE gene promoter region contains NFκB binding sites NFκB activation could increase the RAGE mRNA expression [35]. In addition to the fact that binding of AGE to RAGE activates NFκB, TNF-α activates RAGE expression through NFκB activation [36]. These may suggest a self-perpetuating cycle among AGE, RAGE and NFκB signalling, and cytokines. Moreover, TNF-α and IL-1 have the ability to promote the synthesis and release of matrix metalloproteinases [5].

The enhanced release of AGE-modified molecules during cartilage degradation into the synovium and the activation of FLS through AGE-RAGE interaction may initiate inflammatory responses. In RA, activated NFκB in FLS is involved in the regulation of inflammatory cytokines, adhesion molecules and matrix-grading enzymes, but it also mediates resistance of FLS against apoptosis [37]. Thus, the observation that AGE-BSA failed to induce apoptosis in FLS from OA patients and rather mediates necrosis may also be caused by AGE-induced NFκB activation.

**Figure 14:**
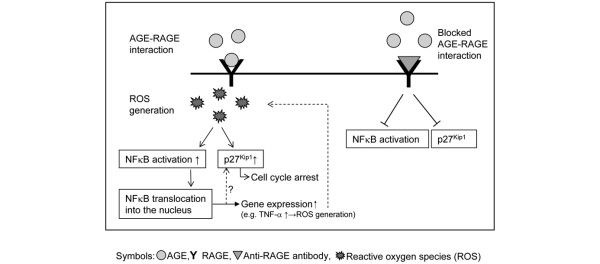
Hypothetical relation between AGE-induced NFκB activation and cell cycle arrest in osteoarthritic FLS. Reactive oxygen species (ROS), known to be induced by receptor for AGEs (RAGE) activation, could be a common denominator for p27^Kip1 ^induction and NFκB activation. In turn, nuclear factor kappa B (NFκB) activation results in enhanced TNF-α expression and TNF-α-induced ROS generation, which may form a vicious loop. NFκB may also directly influence p27^Kip1 ^expression.

We showed that the AGE-RAGE interaction in FLS cells is leading to NFκB activation and on the other hand is increasing the expression of p27^kip1^. Furthermore, both events are inhibited when RAGE-neutralising antibody was added to the cells prior to AGE-BSA addition. Moreover, the activation of NFκB upregulates RAGE gene expression itself. Based on our data we can hypothesise that the signaling events induced from AGE-RAGE interaction are activating different signaling pathways in the FLS cells which have likely some crossover points. More experiments that are beyond this contribution are needed to define the molecular interactions between NFκB activation and the regulation of p27^kip1 ^expression. However, some suggestions can be made. It has been previously shown that AGEs increase the formation of reactive oxygen species (ROS) [38]. ROS activate NFκB as well as stimulate p27^Kip1 ^expression [39,40]. Figure 14 shows this hypothetical relation with ROS a common denominator. However, further studies are necessary to test this intriguing hypothesis.

FLS are an integral part of the RANK/RANKL interaction system, which primarily initiates the maturation, activation and stimulation of osteoclasts. Osteophyte formation and subchondral bone sclerosis are evident for disturbed subchondral bone remodelling towards enhanced tissue formation in OA. AGE-BSA reduces the RANKL and osteoprotegerin expression in our study. These findings were specific for FLS from OA patients and was not observed in human dermal fibroblasts. In a recent contribution Wittrant and colleagues report that high glucose levels (25 mM) inhibit RANKL expression and osteoclast differentiation and formation [41]. In addition, AGE-BSA totally inhibited osteoclastogenesis in rabbit and mouse osteoclasts [42]. These findings support our data that AGE-BSA plays an important role in OA.

## Conclusions

In summary, the present study demonstrates that AGEs modulate FLS growth and expression of genes involved in the pathophysiological process of OA. This may reflect a molecular mechanism by which inflammation and tissue degradation in OA continues and leads to functional and structural impairment of the joints.

## Abbreviations

AGEs: advanced glycation end products; AGE-BSA: AGE-modified bovine serum albumin; BrdU: bromodeoxyuridine; BSA: bovine serum albumin; cDNA: complementary deoxyribonucleic acid; CML: N^ε^-carboxymethyllysine; Co-BSA: control-BSA; DMEM: Dulbecco's modified Eagle medium; EMSA: electrophoretic mobility shift assay; ELISA: enzyme-linked immunosorbent assay; FCS: fetal calf serum; FLS: fibroblast-like synovial cells; GAPDH: glyceraldehyde 3-phosphate dehydrogenase; HRP: horseradish peroxidase; IL: interleukin; MTT: 3- [4,5-dimethylthiazol-2-yl]-2,5-diphenyl tetrazolium bromide; NFκB: nuclear factor kappa B; OA: osteoarthritis; PBS: phosphate-buffered saline; PCR: polymerase chain reaction; RA: rheumatoid arthritis; RAGE: receptor for AGEs; RANKL: receptor activator of NFκB ligand; ROS: reactive oxygen species; sRANKL: soluble RANKL; SDS: sodium dodecyl sulphate; TNF-α: tumour necrosis factor alpha.

## Competing interests

The authors declare that they have no competing interests.

## Authors' contributions

SF and GW designed the study. SF, MS, CR and TB performed the experiments. JM and GH provided synovial tissue samples and clinical data. SF drafted the manuscript. GW participated in the interpretation of data, layout, writing, and finalization of the manuscript. All authors read and approved the final version of the manuscript.
